# The Phage T4 Antiholin RI Has a Cleavable Signal Peptide, Not a SAR Domain

**DOI:** 10.3389/fmicb.2021.712460

**Published:** 2021-08-11

**Authors:** Denise Mehner-Breitfeld, Jan Michel Frederik Schwarzkopf, Ry Young, Kiran Kondabagil, Thomas Brüser

**Affiliations:** ^1^Institute of Microbiology, Leibniz Universität Hannover, Hanover, Germany; ^2^Department of Biochemistry & Biophysics, Center of Phage Technology, Texas A&M University, College Station, TX, United States; ^3^Department of Biosciences and Bioengineering, Indian Institute of Technology Bombay, Mumbai, India

**Keywords:** phage lysis, holins, antiholins, lysis inhibition, signal peptide, SAR domain

## Abstract

Holin/endolysin-mediated lysis of phage T4 of *Escherichia coli* is tightly regulated by the antiholins RI and RIII. While regulation by the cytoplasmic RIII plays a minor role, the periplasmic antiholin RI binds tightly to the holin T and is believed to directly sense periplasmic phage DNA from superinfections as a trigger for the inhibition of lysis. RI has been reported to contain a non-cleavable signal peptide that anchors the protein to the membrane. Lysis is believed to be induced at some stage by a membrane depolarization that causes a release of RI into the periplasm without cleavage of the signal anchor. For the current model of phage lysis induction, it is thus a fundamental assumption that the N-terminal trans-membrane domain (TMD) of RI is such a signal anchor release (SAR) domain. Here we show that, in contrast to previous reports, this domain of RI is a cleavable signal peptide. RI is processed and released into the periplasm as a mature protein, and inactivation of its signal peptidase cleavage site blocks processing and membrane release. The signal peptide of RI can also mediate the normal translocation of a well-characterized Sec substrate, PhoA, into the periplasm. This simplifies the current view of phage lysis regulation and suggests a fundamentally different interpretation of the recently published structure of the soluble domains of the RI–T complex.

## Introduction

Lytic phages tightly regulate the lysis of their host cells primarily by controlling the access of phage-encoded muralytic enzymes, the endolysins, to the bacterial cell wall (Cahill and Young, 2019). In Gram-negative bacteria, additional proteins that are termed spanins are required for the disruption of the outer membrane (Young, 2014). Endolysins can be either released *via* holes formed by membrane proteins that are collectively termed “canonical holins”, or they can be secreted by the general secretory (Sec) system and remain inactive as membrane-anchored proteins that can be released and activated upon membrane depolarization by associations of so-called “pinholins” (Young, 2014). As the latter endolysins are anchored to the cytoplasmic membrane *via* signal peptides that can exit the membrane without cleavage, these special signal peptides are termed “signal anchor release” (SAR) domains (Xu et al., 2005). The regulation of lysis timing occurs by controlling the activity of the holins. Timing of lysis can be simply achieved by the regulated formation of holins (canonical holins or pinholins) and their accumulation in the cytoplasmic membrane until a critical density is reached for hole formation and/or membrane depolarization (Ryan and Rutenberg, 2007; Pang et al., 2013). In addition, canonical holins often exist in lytic or lysis-inhibiting isoforms that use different start codons; in these “dual start” systems, the ratio of these isoforms, regulated by control of translational initiation, determines the timing of the onset of lysis (Bläsi and Young, 1996).

In addition to the intrinsic and dual-start timing systems, many holins are regulated by specific antiholins that somehow sense superinfections and then delay lysis. This is thought to be a strategy to increase the chance for lysis in an environment with a lower phage abundance and thus potential higher abundance of non-infected hosts (Ramanculov and Young, 2001a). Phage T4 is the prototype for studies on this lysis inhibition (LIN), in which two antiholins, RI and RIII, are involved in binding the periplasmic and cytoplasmic domains of T, respectively (Ramanculov and Young, 2001a; Chen and Young, 2016). The cytoplasmic antiholin RIII alone cannot establish a stable LIN and apparently stabilizes the inhibitory effect of the periplasmic RI (Chen and Young, 2016), whereas RI alone is necessary and sufficient for the establishment of LIN (Moussa et al., 2012). Initial biochemical analyses of RI did not detect processing of the RI signal peptide, and a fusion of the RI signal peptide to the mature domain of alkaline phosphatase PhoA was found to result in membrane-attached full-length protein with no signal peptide cleavage detectable (Tran et al., 2007). The authors concluded that RI possesses a SAR domain, just like the endolysins of pinholin/endolysin systems (Tran et al., 2007). This perspective had a fundamental impact on the interpretation of the X-ray structure obtained from the soluble RI domain in complex with the soluble C-terminal domain of holin T (Krieger et al., 2020). Only soluble domains were used for crystallization, and therefore, no trans-membrane domain (TMD) was in the structure. The authors believed that the crystallized soluble RI domain would be normally membrane-anchored by its assumed SAR sequence, and therefore, the holin-antiholin structure was oriented so that the SAR domain of RI would contact and embed in the membrane (Krieger et al., 2020).

Here, we report further studies on RI, which unambiguously demonstrate that in secretion to the periplasm, RI undergoes normal processing of its N-terminal signal sequence, rather than undergoing SAR domain-dependent secretion as previously reported (Tran et al., 2007). We discuss the significant impact of this finding on the mechanism and structural basis of LIN by RI.

## Materials and Methods

### Strains and Growth Conditions

*Escherichia coli* strain ER2566 (B F^–^λ^–^ *fhuA2* [*lon*] *ompT lacZ*::T7.*1 gal sulA11* Δ(*mcrC-mrr*)*114*::IS*10* R(*mcr-73*::miniTn*10*)(Tet^S^)2 R(*zgb-210*::Tn*10*)(Tet^S^) *endA1* [*dcm*]; NEB, Ipswich, MA, United States) was used for fractionation studies when pCOLA-derived plasmids were used, and strain CC118 (Δ(*ara leu*)7697 Δ*lacX74*Δ*phoA20 galE galK recA1 rpsE argE*_amb_ *rpoB thi*; Lee and Manoil, 1994) was used for PhoA activity assays and for fractionation studies when pBW-derived plasmids were used. *E. coli* XL1-Blue Mrf’ Tet (Stratagene, La Jolla, CA, United States) or DH5α were used for cloning. Cells were grown aerobically in LB medium (1% tryptone, 0.5% yeast extract, and 0.5% NaCl) at 37°C with the appropriate antibiotics (100 μg/ml ampicillin, 50 μg/ml kanamycin). For subcellular fractionations, all cultures were normalized to an OD_600_ of 1.0. Cultures of strains with pCOLA-derived plasmids were harvested after 2-h growth with 1 mM of IPTG added at an OD_600_ of 0.5. Cultures of strains with pBW-derived plasmids were harvested after 1.5-h growth with 0.01% (w/v) rhamnose added at an OD_600_ of 0.5.

### Genetic Methods and Plasmids

The antiholin-gene *rI* was amplified from T4 phage DNA by PCR with the primer pair *rI-Nde*I-F (5′-ATG TAC ATA TGG CCT TAA AAG CAA CAG-3′) and *rI-Bam*HI-R (5′-ATG TAG GAT CCT TCA GTC TCC AAT TTA ATG TTC ATA-3′) and cloned in the corresponding sites of pBW-*tatA*-H6 (Berthelmann et al., 2008). The vector pCOLA-*rI*-HA, used for IPTG-induced expression of *rI* with a C-terminal HA-tag, was generated by cloning the *Nco*I-*Hin*dIII digested *rI*-HA amplified PCR product (*rI-Nco*I-F 5′-ATA TAC CAT GGG CGC CTT AAA AGC AAC AGC AC-3′ and *rI-Bam*HI-HA-TAA-*Hin*dIII-R ATA TAA AGC TTT TAG GCG TAG TCC GGC ACG TCG TAC GGG TAG GAT CCT TCA GTC TCC) into the corresponding sites of pCOLADuet-1 (Novagen, Merck KGaA, Darmstadt, Germany). Single amino acid exchange in the signal peptide cleavage site of RI was introduced by QuikChange^TM^ mutagenesis (Stratagene) of pCOLA-*rI*-HA using the forward primer *rI*-pro-F 5′-GTT TTA TCT CCA TCG ATT GAA CCG AAT GTC GAT CCT CAT TTT G-3′ in conjunction with the reverse primer that covers the identical sequence region. For the fusion of the complete signal peptide of RI or the SAR domains of Lyz from P1 or R from P21 to the mature domain of PhoA, DNA encoding these domains was amplified by template-free PCR using overlapping primers (i.e., *rI*-sp-*Nde*I-F 5′-TAT ATC ATA TGG CCT TAA AAG CAA CAG CAC TTT TTG CCA TGC TAG GAT TGT C-3′, *rI*-sp-*Bam*HI-R 5′-TAT ATG GAT CCG CGA CAT TCG CTT CAA TCG ATG GAG ATA AAA CAA ATG ACA ATC C-3′, *lyz*-sar-*Nde*I-F 5′-TAT ATC ATA TGA AGG GAA AAA CAG CCG CAG GAG GCG GTG CAA TTT GCG CTA TCG CG-3′, *lyz*-sar-*Bam*HI-R 5′-TAT ATT GGA TCC GCC ACA TTG CCA TTA CCC ATT ACG ATG GTA ATC ATC ACC GCG ATA G-3′, *r*-sar-*Nde*I-F 5′-TAT ATC ATA TGC CTC CAT CAT TAC GAA AAG CCG TTG CTG CTG CTA TTG GTG-3′, *r*-sar-*Bam*HI-R 5′-TAT ATG GAT CCG CCA CTG ATG CTA TAG CAA TTG CTC CGC CAC CAA TAG C-3′) and cloned in the corresponding sites of pEXH5-*tac-Bam*HIsp with a *Bam*HI restriction site behind the signal peptidase cleavage site (Richter and Brüser, 2005). The resulting plasmids were named pEX-*rI*-sp-*mat-hip-tac*, pEX-*lyz*-SAR-*mat-hip-tac*, and pEX-*r*-SAR-*mat-hip-tac*. The coding region for the mature domain of HiPIP was exchanged with the corresponding region of PhoA, which was amplified from genomic DNA by PCR with the primer pair *mat-phoA-Bgl*II-F 5′-TAT ATA GAT CTC CGG ACA CCA GAA ATG CCT GTT CTG GAA AAC-3′ and *phoA-Hin*dIII-R 5′-TAT ATA AGC TTG AGC GTA TGC GCC CGT GAT CTG-3′ and ligated with *Bam*HI/*Hin*dIII digested pEX-vectors. In a last step, the three *phoA*-gene fusions were cloned into the *Nde*I/*Hin*dIII digested pBW22 (Wilms et al., 2001), resulting in rhamnose-inducible vectors pBW-*rI*-sp-*mat-phoA*, pBW-*lyz*-SAR-*mat-phoA*, and pBW-*r*-SAR-*mat-phoA*. PCR-amplified DNA encoding full-length PhoA and mature PhoA (*mat-phoA-Nde*I-F 5′-TAT ATC ATA TGC GGA CAC CAG AAA TGC CTG TTC TGG AAA AC-3′, *phoA-Nde*I-F 5′-TAT ATC ATA TGA AAC AAA GCA CTA TTG CAC TAT TGC ACT GGC ACT CTT AC-3′, *phoA-Hin*dIII-R 5′-TAT ATA AGC TTG AGC GTA TGC GCC CGT GAT CTG-3′) were used as positive and negative controls and cloned into the above-mentioned backbone using the *Nde*I/*Hin*dIII restriction sites.

### Biochemical Methods

Subcellular fractionations into periplasm, membranes, and cytoplasm were based on an established osmotic shock protocol (Taubert et al., 2015). Briefly, cells of 50 ml exponentially growing cultures (at OD_600_ = 1.0) were sedimented by centrifugation (3,260 × *g*, 4°C, 10′); resuspended and equilibrated for 10 min in 20 ml of 20% sucrose/10 mM Tris–HCl, pH 8.0/1 mM EDTA at room temperature; and sedimented again (3,260 × *g*, 4°C, 10′). The sucrose solution was completely removed from the cell pellet, and cells were osmotically shocked by resuspension in 1 ml of ice-cold 5 mM MgSO_4_ and incubated for 20 min. This shock releases the periplasm. Cells were sedimented again (16,060 × *g*, 4°C, 10′), and the supernatant (periplasm) was collected. The pellet was resuspended in 1 ml of 5 mM MgSO_4_, and cells were disintegrated by sonication (sample cooled on ice). Cell debris was removed by low-speed centrifugation (16,060 × *g*, 4°C, 10′), and membranes were separated from cytoplasm by ultracentrifugation (130,000 × *g*, 4°C, 30′) and resuspended in 1 ml of 5 mM MgSO_4_. Sodium dodecyl sulfate–polyacrylamide gel electrophoresis (SDS-PAGE) analysis was carried out by standard methods (Laemmli, 1970). For immunoblots, proteins were semi-dry blotted on nitrocellulose membranes, and blots were developed using antibodies directed against the hemagglutinin (HA) tag (Invitrogen, Carlsbad, CA, United States), PhoA (Rockland Immunochemicals, Inc., Gilbertsville, PA, United States), and YidC (donation of Andreas Kuhn, Stuttgart) using the enhanced chemiluminescence (ECL) system (GE Healthcare, Chicago, IL, United States) for signal detection. Horseradish peroxidase (HRP)-conjugated goat anti-rabbit or goat anti-mouse antibodies (Roth, Karlsruhe, Germany) served as secondary antibodies. Biotin carboxyl carrier protein (BCCP) was detected by HRP-coupled Strep-Tactin (IBA Lifesciences GmbH, Göttingen, Germany).

## Results and Discussion

### RI Antiholin Possesses an N-Terminal Cleavable Signal Peptide and Is Released Into the Periplasm as a Mature Protein

Signal anchor release (SAR) domains are very unusual signal peptides that initiate Sec-dependent translocation of proteins and function as membrane anchors after export; these SAR-anchored proteins are spontaneously released from the membrane at a low rate; but when the membrane is depolarized, they undergo quantitative release (Xu et al., 2004). In case of SAR domain-containing endolysins, such as P1 endolysin, this release activates the muralytic activity and thereby causes cell lysis. In case of SAR domain-containing antiholin RI, it is believed that after release, the liberated SAR domain confers profound functional and proteolytic instability to the antiholin, resulting in holin activation, endolysin release through the holin lesions, and cell lysis (Tran et al., 2007).

**FIGURE 1:**
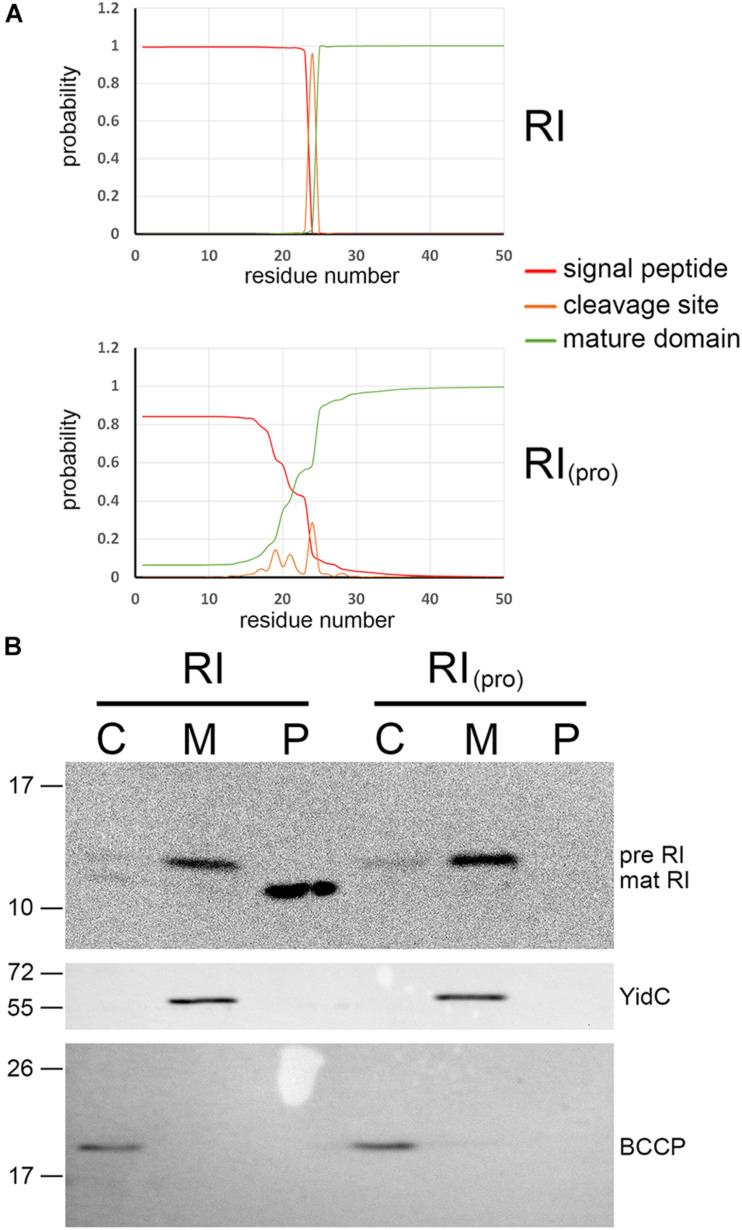
The N-terminal domain of T4 antiholin RI constitutes a signal peptide with an expected signal peptidase cleavage site. **(A)** SignalP comparison of signal peptide from pinholin-associated endolysin P1 (the first identified SAR domain; Xu et al., 2004) with the signal peptide of the T4 antiholin RI. Note the distinct cleavage site likelihood. For more comparisons, see Supplementary Figure 1. **(B)** Transport analysis of RI and a RIpro variant with an inactivated signal peptidase cleavage site (top blot), using subcellular fractionation and sodium dodecyl sulfate–polyacrylamide gel electrophoresis (SDS-PAGE)/Western blotting detection of the hemagglutinin (HA) tag at the C-terminus of RI. The bottom blots are controls that demonstrate no leakage of cytoplasm into the periplasmic fraction [biotin carboxyl carrier protein (BCCP), a cytoplasmic marker] and no leakage of cytoplasmic membranes into the periplasmic fraction (YidC control). C, cytoplasmic fraction; M, membrane fraction; P, periplasmic fraction.

In the course of studies on SAR domains, we initially found that signal peptide prediction by SignalP 5.0 (Almagro Armenteros et al., 2019) strongly differed between RI and the P1 SAR endolysin Lyz (Figure 1A): the N-terminus of Lyz contained no likely signal peptide cleavage site, with only a 40% probability cleavage site after Gly24, whereas RI was predicted to have a very likely cleavage site after Ala24 (95% probability). Other pinholin-associated endolysins such as the SAR endolysin R of phage P21 resembled the P1 endolysin in having no likely cleavage site, and these SAR domains thus clearly differed from the N-terminal domain of RI (Supplementary Figure 1). We therefore re-evaluated the potential SAR domain functionality of RI in *E. coli*. C-terminally HA-tagged RI was recombinantly produced by use of an IPTG-inducible vector system and detected in subcellular fractions (cytoplasm, membrane, and periplasm) by SDS-PAGE/Western blotting analysis (Figure 1B). As no holins were present in this system, SAR domains were expected to result in membrane-anchored full-length protein, with little or no release of full-length protein into the periplasm. However, the RI antiholin was clearly processed, and only mature soluble protein was released into the periplasmic fraction. These data indicated that the signal peptide of RI is cleavable and functioning as expected for a normal soluble periplasmic protein, rather than constituting a SAR domain.

Processing of the RI protein is predicted to occur after an IEA motif, which is not the consensus AXA, in which X can be any amino acid but proline, but still should be acceptable for the signal peptidase LepB, which has been shown to be fully functional with aliphatic side chains at position −3 relative to the cleavage site (Karamyshev et al., 1998). As prolines can abolish cleavage when placed in the cleavage site (Karamyshev et al., 1998), we examined processing and release of mature protein with an RI variant carrying an Ala24Pro mutation (Figure 1B). Notably, processing was blocked and no mature RI was detectable with this variant, indicating that the N-terminus is a standard signal peptide that is cleaved after residue Ala24. In any case, no full-length RI was detectable in the periplasmic fraction, indicating that RI is not released from the membrane without cleavage of its signal peptide. RI therefore possesses a normal cleavable Sec signal peptide to direct its transport into the periplasm. This processing is likely a requirement for LIN, and further studies will hopefully clarify this aspect. In the original report of a SAR domain in RI, the detection of full-length RI in the periplasmic fraction was most likely due to the use of lysozyme/EDTA treatment to generate spheroplasts and a periplasmic fraction, a method notorious for the potential to give cytoplasmic contamination (Tran et al., 2007). Also a contamination by membranes could explain these data. Moreover, no control for cytoplasmic or membrane material in the periplasmic fraction was provided. We used the more reliable osmotic shock protocol, which results in the release of periplasm from the cells without destroying the cell wall (Taubert et al., 2015). In this method, the cytoplasmic membrane is stabilized by 5 mM of MgSO_4_ and the intact cell wall, which prevents leakage of cytoplasm or cytoplasmic membranes into the osmotic shock fraction (the periplasm). Using the BCCP as a cytoplasmic marker protein, we obtained no detectable cytoplasmic contamination in the periplasmic fraction (BCCP in Figure 1B).

### The RI Signal Peptide Can Target the Mature Domain of Alkaline Phosphatase PhoA Into the Periplasm of *Escherichia coli*

**FIGURE 2:**
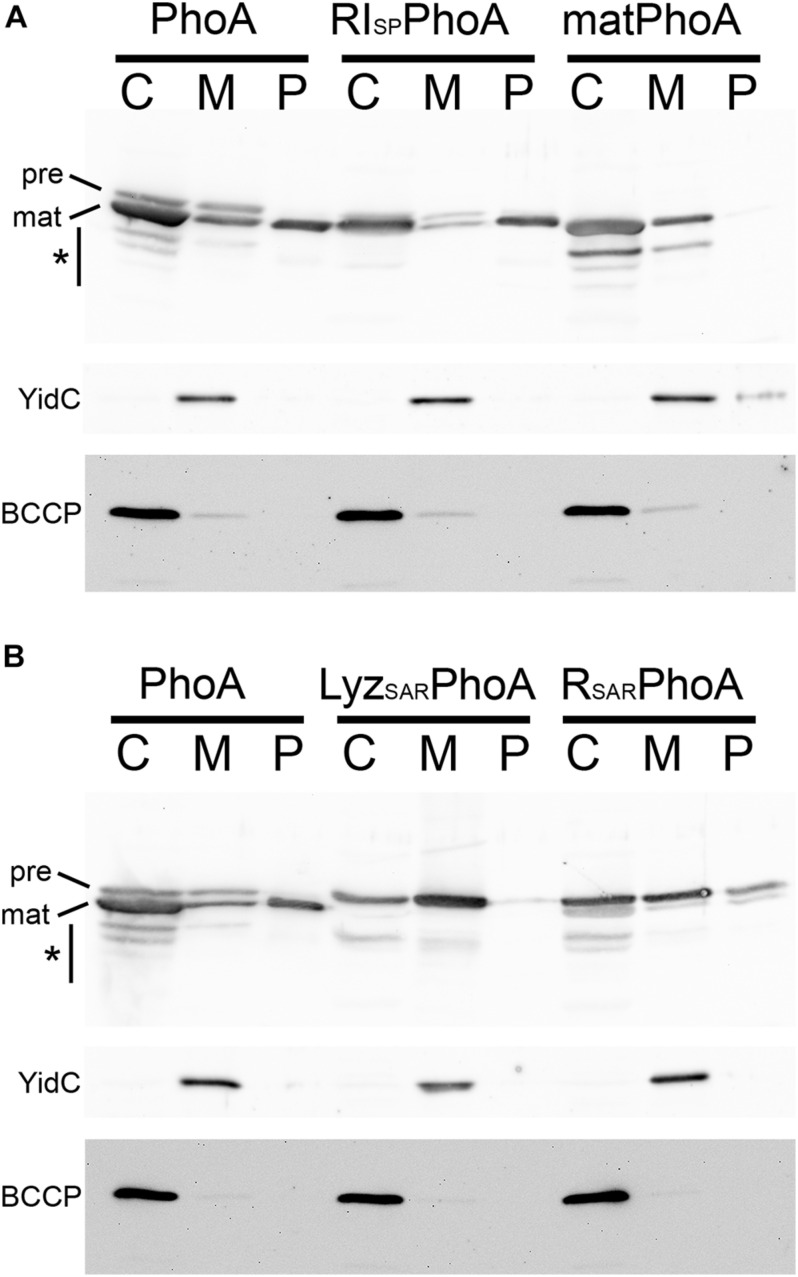
The RI signal peptide can mediate the transport of alkaline phosphatase PhoA into the periplasm. **(A)** Detection of PhoA in subcellular fractions of strains producing either full-length PhoA (PhoA), a RI signal peptide fusion with the mature domain of PhoA (RI_SP_PhoA), or signal peptide-deficient mature PhoA (matPhoA), using SDS-PAGE/Western blotting. **(B)** SDS-PAGE/Western blotting detection of PhoA and the PhoA fusions to the SAR domains of endolysins Lyz (from phage P1) and R (from phage P21). Note that SAR domains of these pinholin-associated endolysins are not efficiently cleaved and the precursor becomes detectable in the periplasmic fraction. PhoA and its derivatives were detected by PhoA-specific polyclonal antibodies. Asterisks indicate regions with degradation bands. BCCP detection showed that no cytoplasm was leaking into the periplasmic fractions. The YidC control monitored potential contamination by cytoplasmic membranes. C, cytoplasmic fraction; M, membrane fraction; P, periplasmic fraction. Asterisks indicate regions with degradation bands.

In the original report of a SAR domain at the N-terminus of RI, the authors confirmed their SAR domain hypothesis by showing that a fusion of the putative SAR domain to the mature domain of alkaline phosphatase, named RI_NTD_ΦPhoA, was also exported without processing (Tran et al., 2007). Inspection revealed that, in this fusion protein, a proline residue was placed immediately adjacent to the signal peptidase cleavage site (+1 position), a position where proline substitutions are known to block signal peptide cleavage (Barkocy-Gallagher and Bassford, 1992; Karamyshev et al., 1998). We therefore analyzed the RI signal peptide–PhoA fusion with its native signal peptidase cleavage site (RI_SP_PhoA) and compared its transport with natural PhoA precursor as positive control (PhoA) and signal peptide-lacking mature PhoA (matPhoA) as negative control (Figure 2). As expected, the RI signal peptide of RI_SP_PhoA was as efficiently cleaved as the natural signal peptide of PhoA, and processed PhoA was thereby released into the periplasm in both cases (the RI_SP_PhoA fusion and PhoA; Figure 2A). Precursor was detectable in the cytoplasmic and membrane fractions, and degradation to mature size and below was detectable, especially in the cytoplasmic fraction, indicating saturation of the transport system and proteolytic cleavages of accumulating PhoA in the cytoplasmic compartment. Cytoplasmic degradation bands are expected, as PhoA cannot fold properly in the reducing cytoplasmic environment (Sone et al., 1997; DeLisa et al., 2003). Degradation of PhoA to mature size is likely due to partial folding of mature domains and degradation of the unfolded signal peptide. Note that this expression system caused more cytoplasmic accumulation than the *rI* expression system (Figure 1). The negative control without a signal peptide remained in the cytoplasm and showed the same degradation bands. A portion of matPhoA was in the membrane fraction, which was likely due to some contamination of membranes by cytoplasm, as shown by detection of the cytoplasmic BCCP control in that fraction.

To directly compare the cleavable RI signal peptide with SAR domains, we analyzed constructs that had fused the respective SAR domains with PhoA (Figure 2B). Notably, while the RI signal peptide was cleaved and mature PhoA was released into the periplasm, the SAR sequences of the two tested pinholin-associated endolysins (from phages P1 and P21) were only released in small quantities, and the majority of the released protein was detected as non-cleaved precursor—in full agreement with the SAR domain function of these endolysins (Xu et al., 2004; Park et al., 2007).

Together, these data demonstrate that the RI signal peptide also functions as cleavable signal peptide when fused to the mature domain of PhoA, and the previous study did not observe this due to the proline residue that was incorporated in the signal peptidase cleavage site in the fusion construct (Tran et al., 2007).

### Implications for the Structural Basis of Lysis Inhibition

**FIGURE 3:**
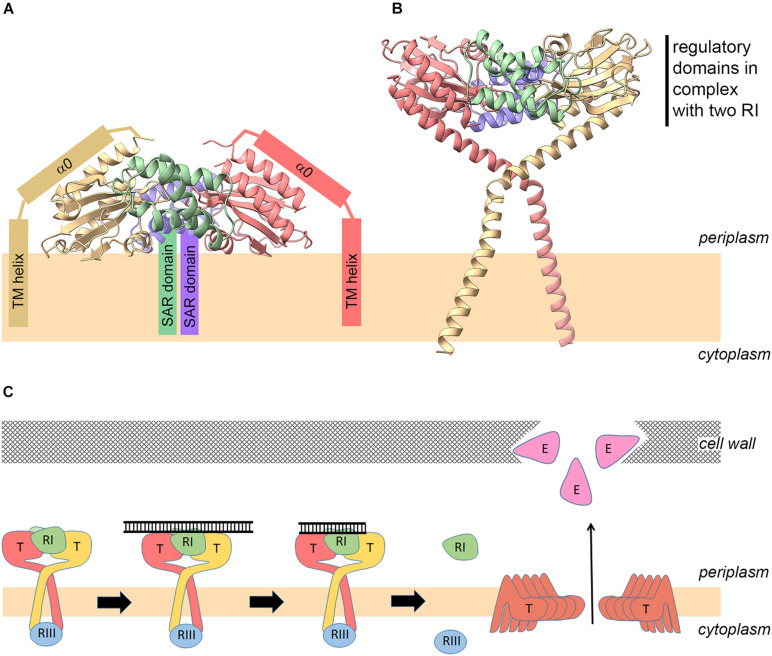
New model for the holin–antiholin complex and revised mechanism of lysis regulation. Model of the holin–antiholin complex as reported previously [**(A)**; Krieger et al., 2020], and as proposed in this study **(B)**, based on the fact that the antiholin N-termini are not membrane-anchored and therefore do not need to be re-oriented back toward the bilayer. **(C)** Revised model for regulation of lysis and lysis inhibition (LIN). (1) Early after infection, the lowly abundant holins form complexes with antiholins. (2) On the periplasmic side, the complex between RI and the regulatory domain of the holin can bind periplasmic DNA of superinfections, which inhibits hole formation even at later higher holin abundance due to stabilization of the RI–T interaction. (3) DNA is degraded over time, and (4) when DNA is not sufficiently stabilizing the RI–T complex anymore at later time points, the highly abundant holins can undergo conformational rearrangements that lead to hole formation, resulting in endolysin release and lysis.

Recently, the structure of the periplasmic domain of the T holin (residues 56–218; T_CTD_) in complex with the putative soluble domain of the RI antiholin (residues 25–92; sRI) has been solved by crystallography (Krieger et al., 2020). It shows that two T_CTD_s bind to two sRIs in a heterotetramer. For protein overproduction and crystallization purposes, the N-terminus of T, including the TMD, was recombinantly removed from the holin construct. Similarly, the reading frame of the RI construct started at position Ala24, which fortuitously corresponds to the signal peptidase cleavage site. Thus, the RI product that was used for crystallization differs from the actual mature, periplasmic RI only by the presence of the N-terminal fMet residue. While the complex structure showed defined electron density for almost the complete RI protein (lacking only two residues at the C-terminus), the holin T structure lacked well-ordered electron density up to position Lys78 (Krieger et al., 2020). As at that time it was believed that the antiholin in this complex should naturally be membrane-anchored by its assumed SAR domain, the whole complex was oriented with the N-termini of the crystallized antiholin RI subunits toward the membrane. In this orientation, the N-termini of the periplasmic holin domains in the complex pointed out, rather than inwards, toward the membrane. To accommodate this orientation, it was proposed that the non-resolved bridging residues might form a kink and a helix that would locate the N-terminus of the periplasmic domain on the same face with the N-termini of the antiholins (Figure 3A). Several aspects of this model seem unsatisfactory. First the observed high flexibility of the bridging residues would be unexpected for helices that are tightly associated with the surface of the folded C-terminal domain. Moreover, there was no evidence at the sequence or electron density level for a sharp kink that would have been a prerequisite for the proposed structure. Finally, the model placed the surface of the crystallized tetramer at the membrane surface, despite the absence of a significant hydrophobic character on the relevant domain of the structure.

The finding that mature RI does not contain a membrane anchor (SAR domain) permits a simpler and more compelling model in which the N-termini of the two holin subunit globular domains are pointing to the membrane (Figure 3B). In this model, the bridging residues connect the globular C-terminal domains to the TMD, which explains the disordered structure of the bridging residues in the crystals in the absence of a membrane anchor (Krieger et al., 2020). Also, the model has the advantage that it does not imply any membrane interaction of the structurally solved globular domains. The exact orientations of the bridge regions and trans-membrane helices in the model shown in Figure 3B certainly are hypothetical. The model serves only to illustrate that in this orientation the N-terminal TMDs of the holin can easily reach the membrane, and the globular domains do not contact the membrane. Such a structure would also inhibit lateral holin multimerization and therefore hole formation. The model has been generated by simple helical extension of the N-termini of the solved tetramer structure to include the bridge region, followed by energy minimization using Chiron (Ramachandran et al., 2011) and addition of the trans-membrane helices. A single kink was introduced at Ser61 in the bridge region that was sufficient for a trans-membrane orientation of the trans-membrane helices. Indeed, there is already some experimental evidence for such an N-terminally extended helix and a kink at Ser61: in one of the crystal structures (6PXE), a continuous helix was seen from Ser92 up to Lys62, and this long helix could be fitted to cryo-electron microscopy (cryo-EM) electron density, in full agreement with the model. The cryo-EM electron density provides no space for an N-terminally further extended helix beyond Ser61 (Krieger et al., 2020), indicating a kink in that region.

The small cytoplasmic N-terminal domain of T, which interacts with the cytoplasmic antiholin RIII (Chen and Young, 2016), has not been included. Despite its lack of detail, the structure serves to illuminate its principle advantages in comparison with the model shown in Figure 3A with respect to the orientation of the N-termini of the subunits and consequently the accessibility of the membrane, the flexibility of the bridging residues, and the required solvent exposure of the globular domains.

### Lysis Inhibition and Lysis Triggering Without a Signal Anchor Release Domain

As RI has no SAR domain, a regulatory role of a SAR domain release in response to membrane depolarization does not need to be implemented anymore in the mechanistic model. This fits well to the observation that the recombinant production of soluble processed RI (using a fusion of the PhoA signal peptide to the C-terminal domain of RI) is fully functional in LIN (Tran et al., 2007). Moreover, the DNA-binding activity of the holin–antiholin complex suggests that recognition of periplasmic DNA from phage superinfection could be a signal. Thus, degradation of the periplasmically located phage DNA could determine the time point after superinfection at which LIN can collapse, allowing the formation of the holin holes (Krieger et al., 2020). It was speculated that the assumed SAR domain needs to be released upon some membrane depolarization, and this would result in a dissociation of the holin–antiholin complex, which in turn would trigger hole formation by free holins and subsequently endolysin release and lysis (Krieger et al., 2020). LIN can be collapsed at any time by treatment with energy poisons (Krieger et al., 2020), and this has been taken as an argument for a SAR domain release as trigger for holin activation. The collapse of LIN has been achieved by cyanide addition (Ramanculov and Young, 2001a), which is known to trigger phage lysis (Doermann, 1952). However, more recent analyses indicate that cyanide does not uncouple energized membranes but rather inhibits uncouplers such as FCCP or DNP (Khailova et al., 2017). Interestingly, this effect appears to be due to an effect of cyanide on membrane protein conformations (Khailova et al., 2017). This changes the interpretation of cyanide effects on phage lysis: cyanide likely affects holin structures in a way that induces hole formation, even if DNA is present in the periplasm. Cyanide effects are therefore not an argument for a role of membrane depolarization in lysis mediated by canonical holins.

These results lead to a simpler model for LIN (Figure 3C). The holin–antiholin complex may form at low holin concentrations already at early stages of the infection and sense DNA from superinfection, which could stabilize this holin–antiholin complex in a way that prevents structural rearrangements of the holin for hole formation, which otherwise would readily occur at higher holin density in the membrane. The kinetics of periplasmic DNA degradation, in conjunction with the synthesis rates of RI and T, would thus determine the time point at which sufficient holin is free to assemble to hole structures. Of course, new superinfections would delay this juncture even further. In this scenario, RI degradation by the periplasmic protease DegP would not play a significant role in holin regulation, which explains the observation that *degP* deletion had no effect on LIN (Tran et al., 2007). Maybe holin-bound RI is more stable than the soluble periplasmic RI, which may form tetrameric structures that have been structurally resolved (Krieger et al., 2020). In the above-described working model (Figure 3C), the two processes “holin accumulation” and “DNA-triggered stabilization of the holin–antiholin complex” are integrated, and the antiholins dissociate from the holin passively due to the structural re-arrangement of the holin at high abundance in the membrane. However, it is still possible that the mechanism might involve some unknown specific trigger for antiholin dissociation, other than holin–holin interactions at high holin concentrations, but the fact that the complex could be crystallized without bound DNA argues against its instability and thus against this hypothesis.

It is important to note that holin-dependent lysis apparently does not require the C-terminal domain, as an amber mutation at position Gln87 still permits holin functionality (Ramanculov and Young, 2001b). It therefore appears that the relevant function of the C-terminal domain of the holin is, in conjunction with the mature RI protein, to serve as a DNA sensor for LIN under superinfection conditions, which is why the RI mutations—as well as the RV mutations that are mutations in the holin itself—all selectively inactivate the process of DNA sensing and LIN.

## Data Availability Statement

The original contributions presented in the study are included in the article/Supplementary Material, further inquiries can be directed to the corresponding author/s.

## Author Contributions

DM-B carried out the experiments. JMFS and KK contributed to the genetic work. DM-B, RY, KK, and TB analyzed the data. TB designed and supervised the study and wrote the manuscript. All authors contributed to the final manuscript.

## Conflict of Interest

The authors declare that the research was conducted in the absence of any commercial or financial relationships that could be construed as a potential conflict of interest.

## Publisher’s Note

All claims expressed in this article are solely those of the authors and do not necessarily represent those of their affiliated organizations, or those of the publisher, the editors and the reviewers. Any product that may be evaluated in this article, or claim that may be made by its manufacturer, is not guaranteed or endorsed by the publisher.
