# Supplementary figures and images for: The Phage T4 Antiholin RI Has a Cleavable Signal Peptide, Not a SAR Domain
Source: Front Microbiol. 2021 Aug 11;12:712460. doi: 10.3389/fmicb.2021.712460 (PMC8385771; doi:10.3389/fmicb.2021.712460)

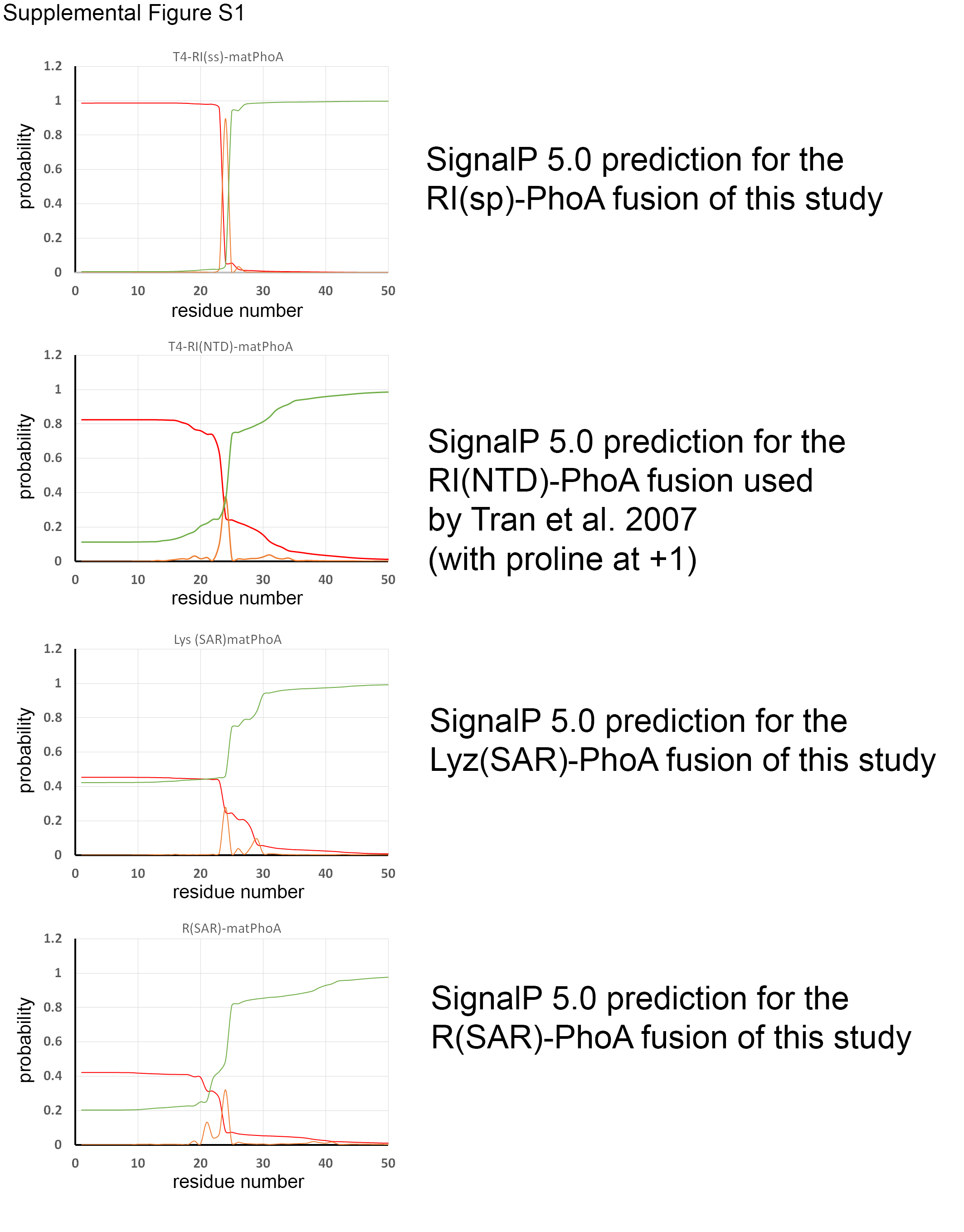

Supplement: Supplementary file 1 [file Image_1.TIF]
